# Retraction Note to: Gonadotropin releasing hormone analogue (GnRHa) alters the expression and activation of Smad in human endometrial epithelial and stromal cells

**DOI:** 10.1186/s12958-015-0016-1

**Published:** 2015-04-03

**Authors:** Xiaoping Luo, Jingxia Xu, Nasser Chegini

**Affiliations:** Department of Obstetrics and Gynecology, University of Florida, Gainesville, Florida USA

## Retraction

This article [1] has been retracted by the Editor following an investigation by the Office of Research at the University of Florida. The investigation confirmed that the data presented in Figures 1, 2, 3A, 3B, 5, 6 and 7 have been fabricated or falsified by the last author.
